# Dynamic DNA Energy Landscapes and Substrate Complexity in Triplet Repeat Expansion and DNA Repair

**DOI:** 10.3390/biom9110709

**Published:** 2019-11-06

**Authors:** Jens Völker, G. Eric Plum, Vera Gindikin, Kenneth J. Breslauer

**Affiliations:** 1 Department of Chemistry and Chemical Biology, Rutgers University, Piscataway, NJ 08854, USA; jvolker@chem.rutgers.edu (J.V.); vgind@chem.rutgers.edu (V.G.); 2 IBET Inc, Columbus, OH 43220, USA; plumge@gmail.com; 3 The Rutgers Cancer Institute of New Jersey, New Brunswick, NJ 08901, USA

**Keywords:** DNA energy landscapes, differential scanning calorimetry (DSC), dynamic DNA states, triplet repeat expansion (TRE), abasic site lesion, 8oxoG lesion, base excision repair (BER)

## Abstract

DNA repeat domains implicated in DNA expansion diseases exhibit complex conformational and energy landscapes that impact biological outcomes. These landscapes include ensembles of entropically driven positional interchanges between isoenergetic, isomeric looped states referred to as rollamers. Here, we present evidence for the position-dependent impact on repeat DNA energy landscapes of an oxidative lesion (8oxodG) and of an abasic site analogue (tetrahydrofuran, F), the universal intermediate in base excision repair (BER). We demonstrate that these lesions modulate repeat bulge loop distributions within the wider dynamic rollamer triplet repeat landscapes. We showed that the presence of a lesion disrupts the energy degeneracy of the rollameric positional isomers. This lesion-induced disruption leads to the redistribution of loop isomers within the repeat loop rollamer ensemble, favoring those rollameric isomers where the lesion is positioned to be energetically least disruptive. These dynamic ensembles create a highly complex energy/conformational landscape of potential BER enzyme substrates to select for processing or to inhibit processing. We discuss the implications of such lesion-induced alterations in repeat DNA energy landscapes in the context of potential BER repair outcomes, thereby providing a biophysical basis for the intriguing in vivo observation of a linkage between pathogenic triplet repeat expansion and DNA repair.

## 1. Introduction

**Figure 1:**
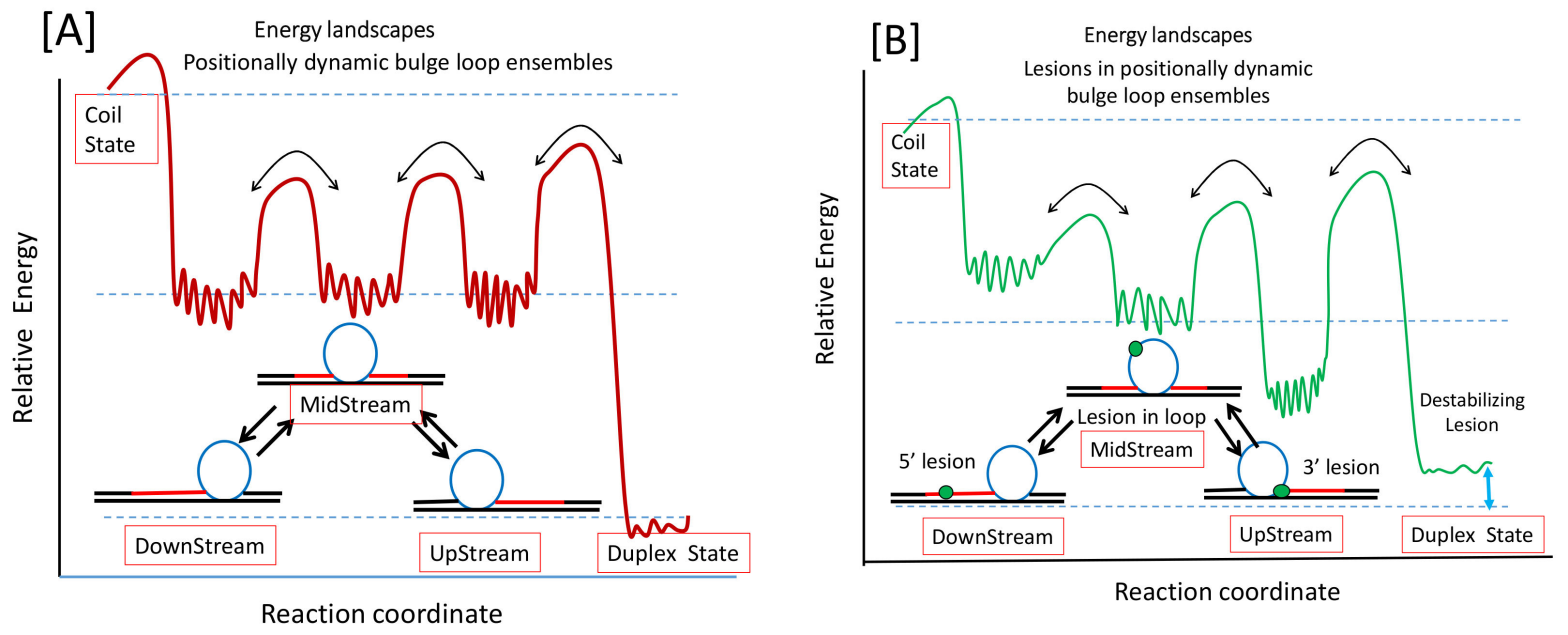
A schematic representation of the dynamic triplet repeat energy landscape in the absence (**A**) and presence (**B**) of a DNA lesion. The fine structure indicated by the squiggly lines within the wells of the rollamers reflects that each bulge loop positional isomer is itself comprised of ensembles of microstates, as we have previously demonstrated [59].

Sequence repeat DNA domains exhibit enhanced propensities to undergo expansion and deletion events in vivo, frequently with deleterious outcomes for the affected organism [1,2,3,4,5,6,7,8]. Such expansion or deletion events often correlate with complex repeat DNA conformational landscapes that can cause dysregulation of common DNA metabolic processes [9,10,11,12,13,14,15,16,17]. In particular, expansion of CNG (N = A,T,C or G) triplet repeat DNA domains in response to DNA replication [13,18,19,20], recombination [21,22], or repair processes [14,23,24,25,26] has been linked to the propensities of these repeats to adopt stable and metastable bulge loop self-structures in competition with the Watson-Crick duplex [27,28,29,30,31,32,33,34,35,36,37,38,39,40,41]. Triplet repeats also exhibit a threshold phenomenon whereby the presence of a stretch of more than 35 or so CNG repeats inevitably leads to pathogenic DNA expansion events [42,43]. We have shown that a bulge loop structure comprised of a part of a larger repeat sequence domain can exist as several isoenergetic loop isomers called rollamers in dynamic equilibrium with each other [44]. Such loop formation and migration results in a complex dynamic energy landscape, illustrated schematically by the red curve in Figure 1A. The three isoenergetic local minima relative to the global minimum of the corresponding fully bonded duplex state reflect each of the three rollameric positional isomers shown schematically in the absence of a lesion in Figure 1A, which creates substrate complexity for the processing of such rollamer domains. This degenerate distribution of isoenergetic rollameric states potentially provides an entropic explanation for the observed threshold phenomenon for DNA expansion [43,44]. In short, rollamers are fleeting/transient dynamic substrates for DNA processing machineries that are optimized to process double helical substrates [26,45,46], thereby potentially explaining why triplet repeat DNAs are prone to undergo expansion and deletion events in vivo.

This substrate complexity becomes even more challenging when the triplet repeat domain contains DNA lesions that require processing within this dynamic landscape by different repair pathways. We have shown that a particular lesion almost always destabilizes the duplex state [47,48,49], but it can either stabilize, remain neutral, or destabilize the static repeat bulge loop state depending on the position of the lesion with respect to the loop domain [50]. Such position-dependent differential impact of lesions on static repeat bulge loops is also expected to significantly influence the properties of dynamic bulge loop rollamer substrates. This next level of lesion-induced substrate complexity is illustrated schematically by the green curve in Figure 1B. Note that the presence of a lesion (indicated by a green dot) can differentially disrupt the isoenergetic degeneracy of the rollameric isomers in a position-dependent manner, as reflected by the example of different well depths for each rollamer shown in Figure 1B.

Below, we experimentally map the impact of DNA lesions embedded within and modulating the complex, dynamic triplet repeat landscape. One goal of these studies is to establish a better appreciation for the dynamic complexities of the repeat DNA substrates encountered by DNA repair pathways during lesion repair, which is part of our broader goal of correlating biophysical properties with biological mechanisms and outcomes. Specifically, we focused on the role of 8oxodG and abasic site lesions as the most common oxidative DNA damage and universal intermediate in base excision repair (BER) of oxidative DNA damage [51,52,53], respectively. Such constructs are biologically relevant since it has been estimated that between 10,000 and 30,000 incidences of oxidative damage to DNA occur in each cell per day [54,55]. Moreover, CAG repeat secondary structures are hotspots for oxidative DNA damage [28,56], while base excision repair (BER) of oxidative DNA lesions within or near CAG repeats leads to DNA expansion in mouse models of Huntington’s disease [57,58]. Here, we present the first experimental evidence in support of such coupling of repair and expansion by mapping the complex, dynamic energy landscapes of potential substrates for DNA processing within dynamic rollamer repeat domains. Specifically, we demonstrate that DNA lesions within or proximal to triplet repeat domains can bias dynamic rearrangement and redistribution of loop isomers in favor of those rollamers where the lesion is positioned to be energetically least disruptive. Our results provide an intriguing biophysical basis for the in vivo observation of the coupling of DNA repair and DNA triplet repeat expansion.

## 2. Materials and Methods

### 2.1. Materials

Oligonucleotides were synthesized in our lab on a 10 µmole scale by standard phosphoramidite chemistry using an Äkta DNA synthesizer or were purchased from IDT (Coralville, IA, USA). Oligonucleotides were purified by Dimethoxytrityl –on (DMT-on) and subsequent repeated DMT-off reverse phase high performance liquid chromatography (HPLC), as previously described [32,34,44,50,60]. The purities of the oligonucleotides were assessed by analytical HPLC and ion spray mass spectroscopy, and were found to be pure by analytical HPLC and better than 98% pure by mass spectroscopy. Purified oligonucleotides were dialyzed using dispo-dialyzers with MWCO 500 Da (Spectrum, CA) against at least two changes of pH 6.8 buffer containing 10 mM cacodylic acid/Na-cacodylate, and 0.1 mM Na2 EDTA and sufficient NaCl to yield a final concentration of 100 mM in Na+ ions. Molar extinction coefficients of the parent DNA oligomers lacking repeats ([CAG]0, [CTG]0) were determined by phosphate assay under denaturing conditions (90 °C) [61,62] and were found to be: ε[CAG]0 (260 nm, 90 °C) = 190,400 M^−1^ cm^−1^; ε[CTG]0 (260 nm, 90 °C) = 186,200 M^−1^ cm^−1^. For all other oligonucleotides, extinction coefficients were determined from continuous variation titrations (Job plots) [63] with the complementary parent oligonucleotide, and were found to be: ε[CTG]2 (260 nm, 90 °C) = 221,700 M^−1^ cm^−1^; ε[CTG]4 (260 nm, 90 °C) = 271,100 M^−1^ cm^−1^. For the abasic site and 8oxodG lesion containing oligonucleotides extinction coefficients were found to be: ε[CAG]6-F(n) (260 nm, 90 °C) = 368,400 M^−1^ cm^−1^; and ε[CAG]6-O(n) (260 nm, 90 °C) = 368,400 M^−1^ cm^−1^ regardless of lesion positions. As expected, for the 40-mers, the impact of a single abasic site or 8oxodG lesion in place of guanine is independent of lesion position and too small to result in a measurable change in extinction coefficient when compared to the [CAG]6 parent 40-mer.

### 2.2. Differential Scanning Calorimetry (DSC) Studies

Differential Scanning Calorimetry (DSC) studies were conducted as previously described using a NanoDSC II differential scanning calorimeter (Calorimetry Science Corporation, Provo, UT, USA) with a nominal cell volume of 0.3 mL [64,65]. Oligonucleotides, at a concentration of 50 µM in strand, were repeatedly scanned between 0 °C and 90 or 95 °C with a constant heating rate of 1 °C /min, while continuously recording the excess power required to maintain sample and reference cells at the same temperature. After conversion of the measured excess power values to heat capacity units (cal/K) and subtractions of buffer vs. buffer scans, the raw DSC traces were normalized for DNA concentration and analyzed using Origin software. The calorimetric enthalpy change (ΔHcal) was derived by the integration of the excess heat capacity curve, and ΔCp was derived from the difference in the linearly extrapolated pre- and post-transition baselines at Tm. ΔS was derived by ΔH/Tm, assuming “pseudomonomolecular” dissociation behavior in which propagation dominates initiation [66]. The Tm is defined as the temperature at the midpoint of the integrated excess heat capacity curve for a given conformational transition, which corresponds to half the sample being denatured for a process that exhibits pseudomonomolecularity.

### 2.3. Modeling of DSC Curves

For our model, we defined six accessible states: the three rollamer isomers (Loop Upstream, Loop MidStream, and Loop Downstream), two states wherein only the non-repetitive terminal upstream or downstream segments and the two proximate CAG triplets are base paired, and the fully denatured reference state. We assume that each triplet acts as a unit and that all unmodified triplets are thermodynamically indistinguishable. A segment containing a lesion within it is independently parameterized. The heat capacity change associated with each segment’s disruption is assumed to be zero.

We derived a partition function for this system parameterized in terms of temperature dependent and temperature independent contributions to each segment’s equilibrium constant [67,68]. A term accounting for the contribution of loop formation is also included. Using the partition function, the change in population of each state is calculated as a function of temperature. The contribution of the population changes to the excess heat capacity is calculated at each temperature to produce a calculated DSC curve. The parameters are adjusted to minimize the difference between the measured and calculated DSC curves. To this aim, we wrote a program in Labview using the Levenberg-Marquardt algorithm for the function minimization.

### 2.4. Ultraviolet Absorption Studies

Ultraviolet (UV) spectra and temperature dependent changes in UV absorbance were measured using an AVIV model 400 UV/VIS spectrophotometer (Aviv Biomedical, Lakewood, NJ, USA). Temperature dependent changes in absorbance at 260 nm with a 1 nm bandwidth were recorded with an averaging time of 5 s while the temperature was raised in steps of 0.5 °C with 1 min equilibration time. Oligonucleotide concentrations were 1.5 or 2 µM in strand.

## 3. Results and Discussion

### 3.1. The Systems Studied

**Figure 2:**
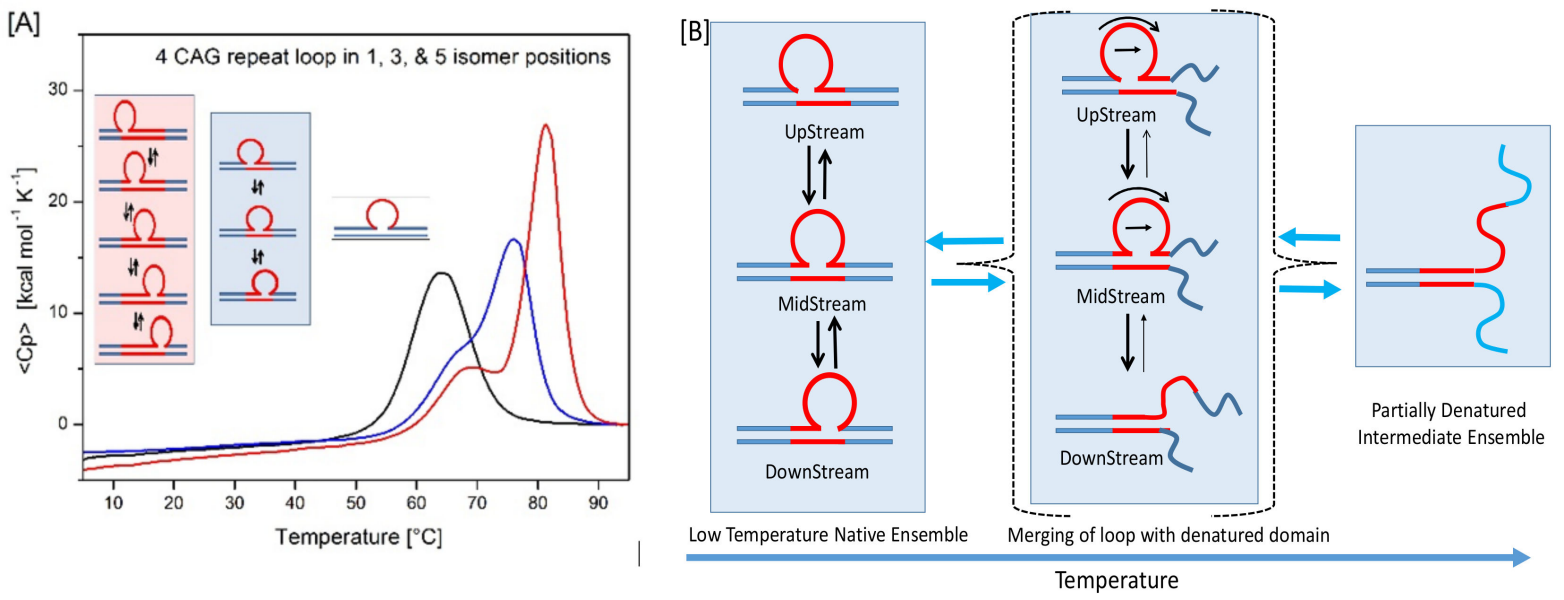
(**A**) Characteristic changes in the excess heat capacity profiles of a static (CAG)4 repeat bulge loop (black) compared to the same (CAG)4 bulge loops existing as three (blue) or five (red) rollamer isomers. The excess heat capacity profile of the five isomer rollamer (red curve), where four additional base pair CAG·CTG triplets can partition between the upstream and downstream duplex domain [44], is included in Figure 2A to demonstrate that the characteristic changes in the shape of the melting curve are general features due to the dynamic nature of the rollamer relative to the static bulge loop construct. (**B**) Schematic presentation of the initial steps in the denaturation pathway for the three isomer (CAG)4 rollamer that is the focus of this paper, reflecting the impact of the dynamic rollamers on the excess heat capacity curves shown in Figure 2A. Denaturation of the residual duplex (the main transition in the DSC thermogram) that is formed as the result of the initial melting step is not shown and conforms to conventional duplex melting behavior.

**Scheme 1:**
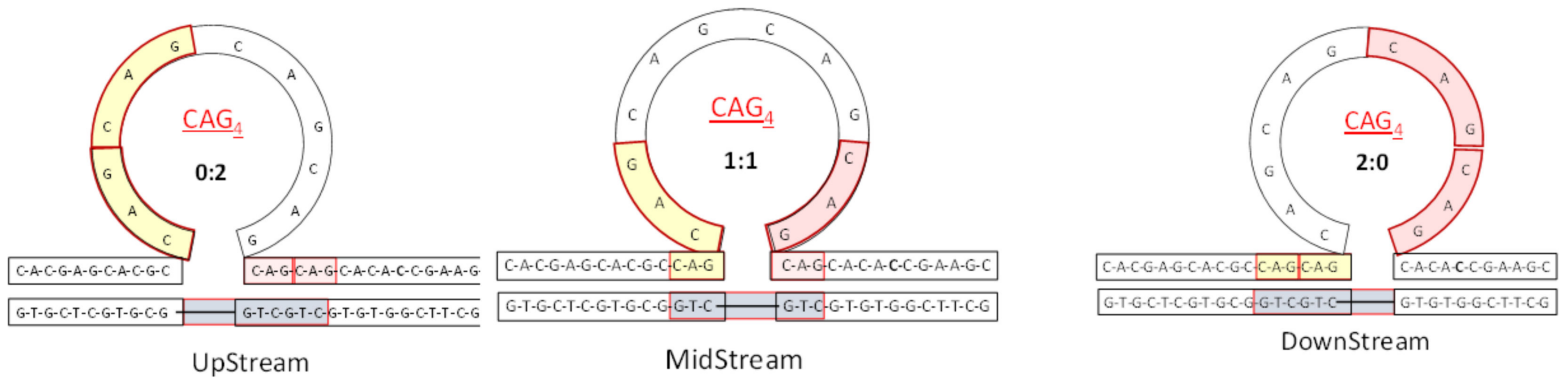
Schematic representation of the three loop isomers (Upstream, MidStream, and Downstream) of our rollamer construct highlighting in color the partitioning of the extra triplets in the upstream (yellow) and downstream (red) domain due to the loop position in each of the isomers. In the UpStream isomer, both additional triplets partition in the downstream duplex domain (0:2); in the MidStream isomer, the additional triplets partition one each in the upstream and downstream duplex domain (1:1); and in the DownStream isomer, both additional triplets partition in the upstream duplex domain (2:0).

We designed a family of rollamer constructs containing a (CAG)4 repeat bulge loop that can exist in three possible isoenergetic loop positions by combining a [CAG]6 oligonucleotide with a partially complementary [CTG]2 oligonucleotide, as per the rules we previously described [44]. As illustrated in Scheme 1, such a construct exhibits a dynamic landscape of 3 interchanging, rollamer bulged loops. This dynamic behavior of (CAG)4 repeat loop rollamers contrasts with the static landscape of the corresponding single, fixed loop structure [32,33,44]. In panel A of Figure 2, note the characteristic changes in the excess heat capacity profiles of a static (CAG)4 repeat bulge loop (black) compared to the same (CAG)4 bulge loops existing as three (blue) or five (red) rollamer isomers. The consequences of such static versus dynamic loop properties are manifest in the differential DSC profiles shown in Figure 2, as described in the figure legend, and elaborated on below.

#### 3.1.1. The Shape of the Rollamer Heat Capacity Curve Reflects the Coupling and Merging of Duplex Arm Melting and Loop Migration

The shape of the excess heat capacity profile of the three isomer rollamer construct shown in Figure 2 is primarily determined by the two additional base paired CAG·CTG triplets not present in the static repeat bulge loop. As shown in Scheme 1, these additional triplet base pairs are dynamically redistributed between the upstream and downstream duplex domains in the three isomers depending on loop position. Due to the relative positions occupied by the loop, we defined each of the three rollamers as loop MidStream with the designation of 1:1, reflective of the two additional triplet base pairs partitioning equally between the upstream and downstream duplex domains; loop UpStream with the designation of 0:2, reflective of both additional triplet base pairs partitioning in the downstream duplex domain; and, loop DownStream with the designation of 2:0, reflective of both additional triplet base pairs partitioning in the upstream duplex domain.

As we previously have shown in the absence of lesions [44], in our construct, denaturation of the rollamer in Figure 2A is initiated by melting of the less stable downstream duplex arm, resulting in a partially denatured complex (the initial transition seen in Figure 2A and the schematic shown in Figure 2B). Denaturation of this downstream domain induces loop migration to merge the loop with the denatured domain in those isomers where the loop is located away from the denatured arm. This process disrupts existing CAG·CTG pairs ahead of the migrating loop while regenerating an equivalent number of CAG·CTG pairs behind the migrating loop. Thus, loop migration is energetically neutral, but kinetically relatively slow on the timescale of our experiments, thereby impacting the shape of the DSC pre-transition peak. As a consequence, the two additional base paired CAG·CTG triplets partition into and stabilize the upstream duplex arm, giving rise to the main transition seen at higher temperature in the excess heat capacity profile. The presence of the free ends (“end effects”) in these oligonucleotides enhances the difference in the melting temperature of the two stable duplex domains, thereby allowing a clear differentiation of the sequential melting steps. The increase in enthalpy and melting temperature in the rollamers compared to the static structure reflects the additional base pairs in rollamers as well as the gain in entropy due to the degeneracy in the loop positions [44].

To summarize, denaturation initiates at the more unstable duplex domain coupled with the enthalpically neutral, but kinetically relatively slow migration of the loop from its three possible positions to merge with the denatured domain. As a consequence, loop migration adds base pairs to the more stable (upstream) duplex domain, thereby resulting in the thermal and thermodynamic stabilization of the upstream domain. Subsequent denaturation of the upstream domain at higher temperatures conforms to conventional oligonucleotide duplex behavior.

#### 3.1.2. Differential Impact of Lesions on Static Versus Dynamic Loop Landscapes

**Figure 3:**
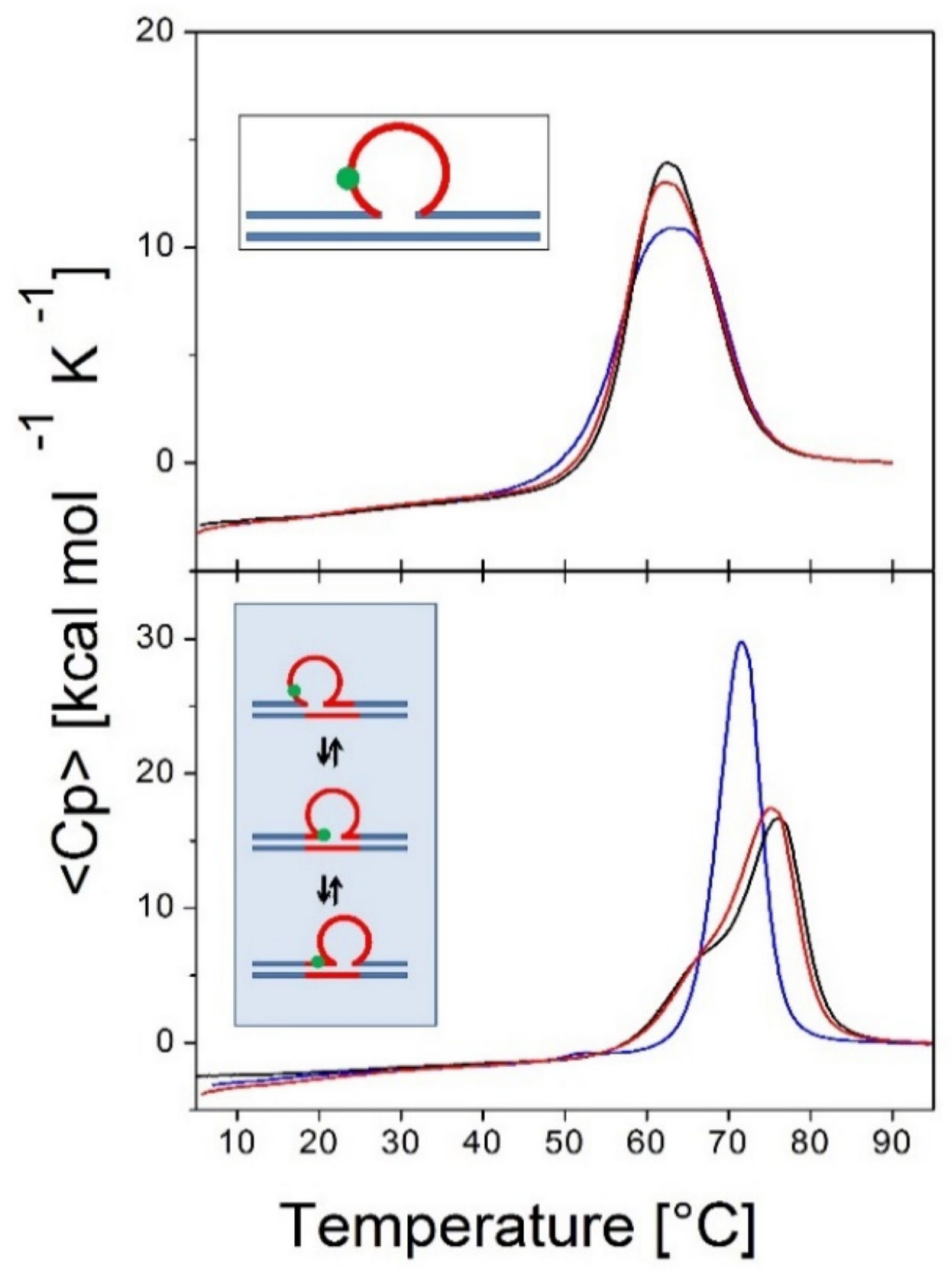
Figure 3 shows the excess heat capacity curves measured for a static (CAG)6 repeat bulge loop (top panel) [50] and dynamic (CAG)4 repeat bulge loop (bottom panel) in the absence (black curve) and presence of either 8oxodG (red curve) or an abasic site lesion (blue curve) in place of dG in the first CAG repeat. Note that whereas the abasic site lesion in the static bulge loop is associated with a slight broadening of the melting transition, but no net change in enthalpy change, it results in the exact opposite melting behavior in the dynamic rollamer construct. In the dynamic rollamer, the abasic site lesion causes the loss of the pre-transition and a significant apparent increase in cooperative melting transition relative to the lesion free rollamer, reflecting lesion induced changes in loop isomer distribution. The excess heat capacity curves therefore provide a good window into the impact of lesions on dynamic loop distributions. As reported for duplex DNA [69,70,71], the impact of 8oxodG lesions in either the static bulge loop or dynamic rollamer is minor.

**Figure 4:**
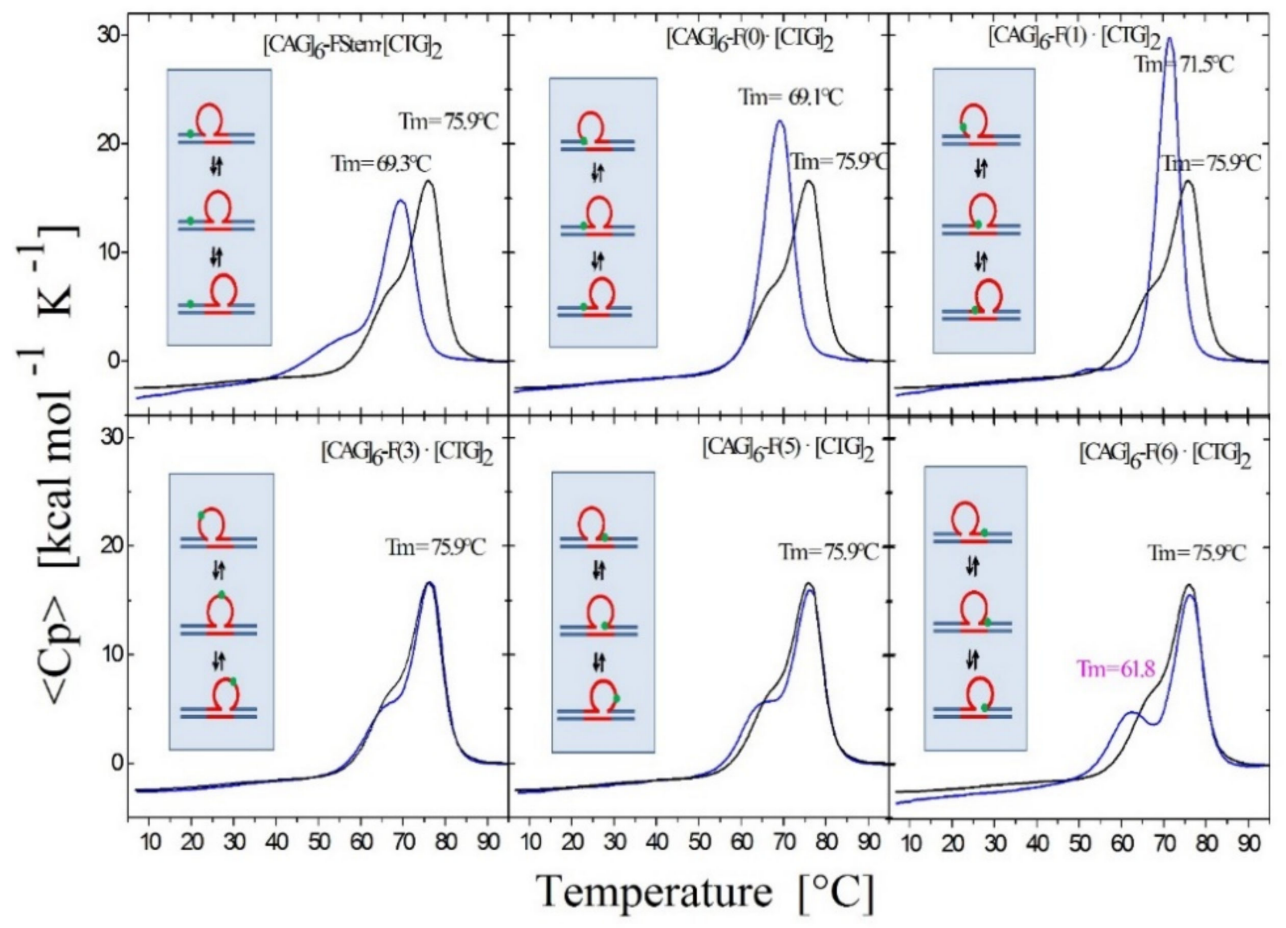
Excess heat capacity curves measured for the [CAG]6-F(n)·[CTG]2 constructs (F = Tetrahydro Furan abasic site analogue) for different lesion positions within the repeat domain F(n), where F replaces the dG in the nth CAG repeat counting from the 5′ end. The lesion free parent construct (black) is the same in all panels, blue curves correspond to abasic site (F) lesions in the CAG strand. A schematic representation of the different lesion position relative to the three possible loop isomers (green dot) is shown as the insert in each panel.

To probe lesion-induced alterations in the triplet repeat dynamic energy landscape, we systematically introduced a single oxidative lesion (8oxodG or abasic site analogue) in place of dG at select positions within the CAG strand (shown schematically in Figure 1) such that multiple possible lesion positions are represented relative to the three rollamer loop isomers (see inserts in Figure 3 and Figure 4 and Table 1). Changes in loop distribution caused by the presence of a lesion in a particular repeat are expected to be reflected in corresponding changes in the excess heat capacity profile during melting. These changes would be due to the altered distribution of base paired CAG·CTG triplets between the upstream and downstream duplex domains, in addition to the position-dependent differential enthalpy effect of the lesion itself. These expectations are borne out by the experimental data. For example, as shown in Figure 3, and noted in the legend, if a lesion is located in the first CAG repeat, we observed that the presence of the lesion within the repeat significantly altered the characteristic shape of the rollamer heat capacity profile relative to the lesion-free parent rollamer, while barely altering the excess heat capacity curve of the static repeat loop construct.

Specifically, whereas the abasic site lesion (blue curve in Figure 3) in the static bulge loop is associated with a slight broadening of the melting transition, but no net enthalpy change [50], it results in the exact opposite melting behavior in the dynamic rollamer construct. In the dynamic rollamer, the lesion causes the apparent loss of the pre-transition and a significant apparent increase in cooperative melting transition relative to the lesion free rollamer, reflecting primarily lesion-induced changes in loop isomer distribution.

The excess heat capacity curves therefore provide a good window into the impact of lesions on dynamic loop distributions. In contrast, the impact of 8oxodG lesions (red curve in Figure 3) in either static bulge loop or dynamic rollamer is minor, with only a small decrease in Tm of the main transition in the rollamer construct as the main lesion impact. The differential impact of 8oxodG versus the abasic site lesion is reflective of the differential impact of these two lesions on DNA stability [69,70,71]. Based on these observations, we concluded that the repeat bulge loop distribution and redistribution as a function of lesion presence and position can be detected and monitored by differential heat capacity signatures, as measured by scanning calorimetry.

#### 3.1.3. Lesion Impact Is Position Dependent

As shown in Figure 4, for all lesion-containing rollamers investigated, and as elaborated in the figure legend, we found characteristic changes in the shape of the excess heat capacity curves relative to that of the control, lesion-free, set of parent rollamers. Significantly, these characteristic changes were specific for the position of the lesion within the repeat sequence. These position-dependent, lesion-induced changes occurred despite the lesions being located in the same nearest neighbor context in all but one of the constructs. Consequently, our observations reflect lesion-induced alterations in loop isomer distribution and occupancy as well as any coupled events. Importantly, we found the same general trends for 8oxodG (not shown).

Figure 4 shows and Table 1 and Table S2 (Supplementary Material) summarizes the data for all of the lesion-containing constructs studied herein. Note that by strategically locating a lesion within a specific repeat within the three dynamic loop constructs, we were able to define features of and extract data from the DSC profiles that allow us to examine the differential distribution of the lesion into different structural domains accessible to the rollamers (5′ or 3′ stem duplex domain; 5′ or 3′ stem/loop junction; or loop domain). Collectively, these results are characteristic of position-dependent, lesion-induced redistribution of dynamic loop structures; a feature that generates a highly complex landscape of dynamic substrates for DNA processing systems.

#### 3.1.4. Position-Dependent Lesion Impact on Rollamer Distribution

**Scheme 2:**
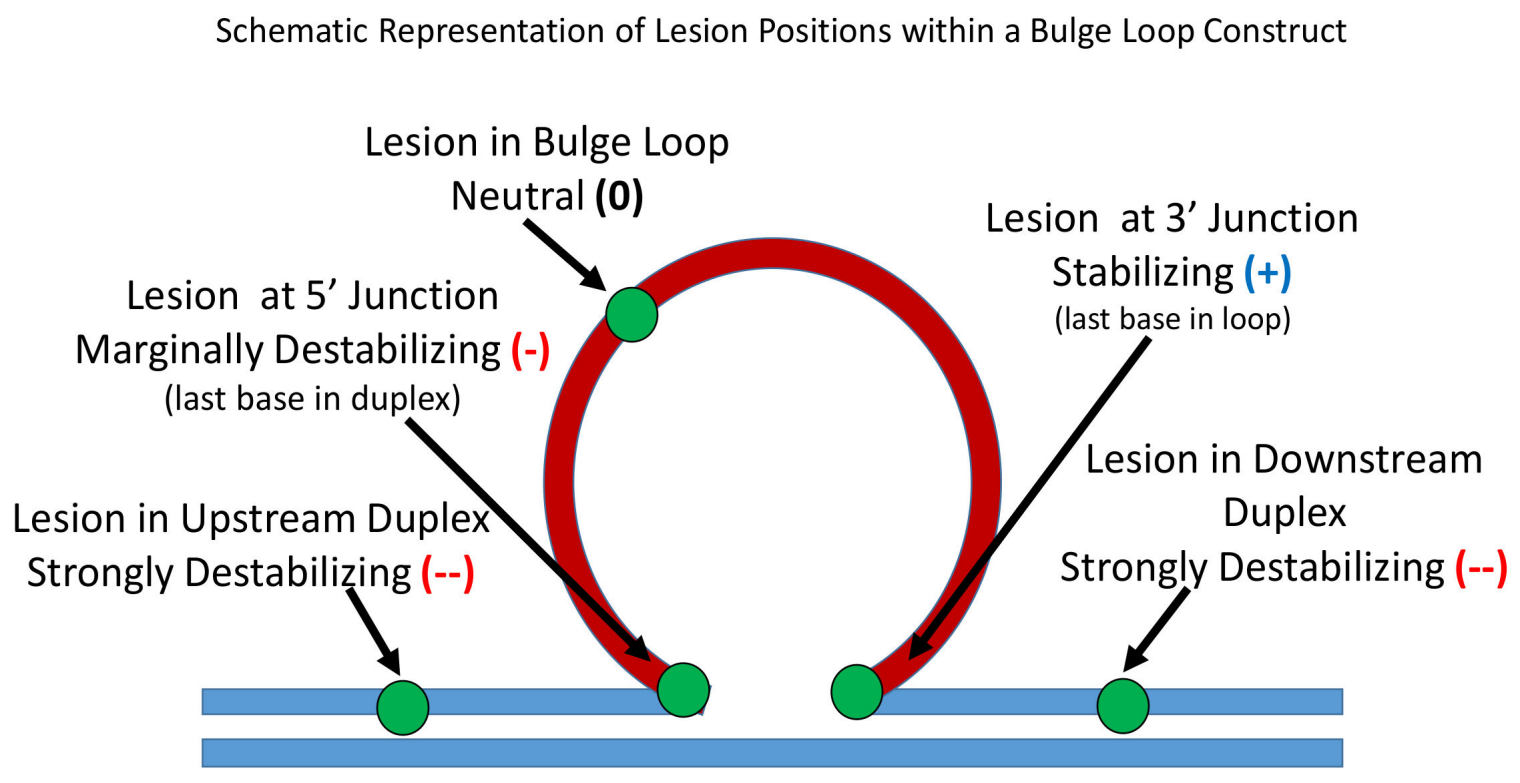
Pictorial map summarizing the position-dependent, differential, enthalpic impacts of lesions within the CAG triplet repeat bulge loops as derived from data on static bulge loops.

To further define the impact of the lesion positions within the dynamic landscape of rollamers, we compared the results reported here with our previous studies on 8oxodG and abasic site lesions in fixed positions within the corresponding, static, single isomer repeat bulge loop [50] (Supplementary Materials). Such comparisons between our datasets on dynamic and static repeat bulge loop constructs allowed us to define a general hierarchy of position-dependent lesion impacts on bulge loop properties. Specifically, relative to the lesion-free, parent bulge loop structure, we found that in static repeat bulge loop constructs, lesions in the duplex portion of the bulge loop structure were strongly enthalpically destabilizing (--); lesions at the 5′ loop-duplex junction were marginally enthalpically destabilizing (-); lesions within the repeat loop domain had no net effect on the enthalpy change (0); whereas lesions at the 3′ junction were overall enthalpically stabilizing (+). Within the bulge loop domain, lesions in the center of a repeat loop may exert a small positive/stabilizing enthalpy contribution. These position-dependent lesion effects are summarized pictorially in Scheme 2.

Direct comparisons of these position-dependent, differential, enthalpic impacts of lesions within the CAG triplet repeat bulge loops, absent error-prone heat capacity adjustments, were justified by the overall similarities in Tm and transition widths for all constructs studied. Significantly, the relative enthalpy trends noted remained unaltered when the heat capacity term was considered. In the constructs studied here, with one exception, the abasic site or the 8oxodG lesion always replaced a Guanine as its redox potential makes guanine the most readily oxidized and therefore most frequently damaged of the DNA bases [72,73]. The single exception in our constructs occurred at the 5′ junction, where F replaced a C instead of a G in the static loops to disrupt the repeat sequence and prevent loop migration. Due to the initial steps of BER repair of 8oxodG lesions [74,75] as well as spontaneous loss of guanines [54], an abasic site results in place of dG. As a consequence of the 5′-3′ directionality and the repeat sequence, the lesion at the 5′ junction occurs in place of the last “formal” base pair of the upstream duplex domain, whereas the lesion at the 3′ junction is the last “formally unpaired” base within the loop domain prior to the beginning of the downstream duplex domain. The relative differences in the enthalpic impact of the lesion at 5′ and 3′ duplex junctions may in part be due to these sequence-induced constraints. The relative differences in enthalpy between lesions at the 5′ and 3 junction may also reflect topological constraints imparted by the three dimensional nature of the bulge loop construct.

Applying these position-dependent criteria to the calorimetric data for the lesion-containing, dynamic rollamers reported here allowed us to assess the lesion impact on rollamer distribution. This information on the impact of lesions on alterations in rollamer distribution is summarized in Table 1. For example, in the construct [CAG]6-F(1)·[CTG]2, (Figure 3 and Figure 4, panel 3) the lesion is located in the first CAG repeat in sequence space, while simultaneously, the lesion may partition into the upstream duplex domain (enthalpically destabilizing), the 5′ loop duplex junction (slightly enthalpically destabilizing), or into the loop (neutral) in conformational space. The observed lack of any enthalpy change relative to the parent construct is consistent with the lesion primarily partitioning into the loop domain; in other words, the population of the rollamer with the lesion located within the loop is enriched relative to the occupancy of the other rollamer conformations. This enthalpy-based conclusion is also consistent with the observed shape of the excess heat capacity curve, as partitioning of a lesion in the first CAG repeat primarily into the loop domain implies that the extra paired CAG base triplets are part of the downstream duplex arm. The resulting increase in melting temperature and enthalpy of the downstream duplex arm causes its melting transition to overlap with that of the upstream domain, thereby resulting in the observed shape of the melting curve.

#### 3.1.5. Lesions Cause Preferential Occupancy of the Enthalpically Most Stabilizing of the Three Possible Loop Isomer States 

In the aggregate, our data show that the lesion causes enriched occupancy of the enthalpically most stabilizing of the three possible loop isomer states; a feature emphasized in Table 1 via yellow highlighting. For example, for the construct [CAG]6-F(6)·[CTG]2 (Figure 4, panel 6), the lesion in the sixth CAG repeat in sequence space may partition in conformational space either into the 3′ loop/duplex junction (enthalpically stabilizing) or the downstream duplex domain (enthalpically destabilizing). The calorimetrically observed more favorable enthalpy relative to the parent rollamer construct is consistent with the lesion preferentially enriching the population of the 3′ loop/duplex junction rollamer. This enthalpy based deduction is also reflected in the shape of the excess heat capacity curve. A partition of the lesion in the last (sixth) CAG repeat into the 3′ loop/duplex junction implies that the two extra base paired CAG·CTG triplets are part of the upstream duplex arm; thereby resulting in an increase in thermal stability and enthalpy of this arm relative to the downstream arm and clearly separated melting transitions. Moreover, if the loop exclusively partitions into the 3′ loop /junction, as implied by the enthalpy gain of this loop position, then denaturation of the downstream arm and loop occur simultaneously in all molecules and no loop migration into the denatured domain occurs. Indeed, the heat capacity curve for [CAG]6-F(6)·[CTG]2 is the only one of those displayed in Figure 4, where both observed melting transitions approximate two- state-like behavior, and where the shape of the pre-transition does not show any distortions due to kinetically controlled loop migration. In summary, our data show that the lesion induces rollamer migration/redistribution to cause preferential occupancy of the enthalpically most stabilizing of the three possible loop isomers, as highlighted in Table 1.

Continuing this straight forward analysis for each of the lesion positions reveals that the presence of the lesion indeed alters the distribution of rollamer isomers in a position-dependent manner through loop migration, as summarized in Table 1. Note that this migration-induced differential distribution of rollamers is defined by partitioning of the lesion into different structural domains (3′ stem/loop junction > loop domains > 5′ stem/loop junction >> 5′ or 3′ stem duplexes), so as to favor those rollamer isomers where the lesion is least energetically disruptive.

### 3.2. Semi-Empirical Modeling Yields Rollamer Distributions Consistent with Our Experimental Results

**Figure 5:**
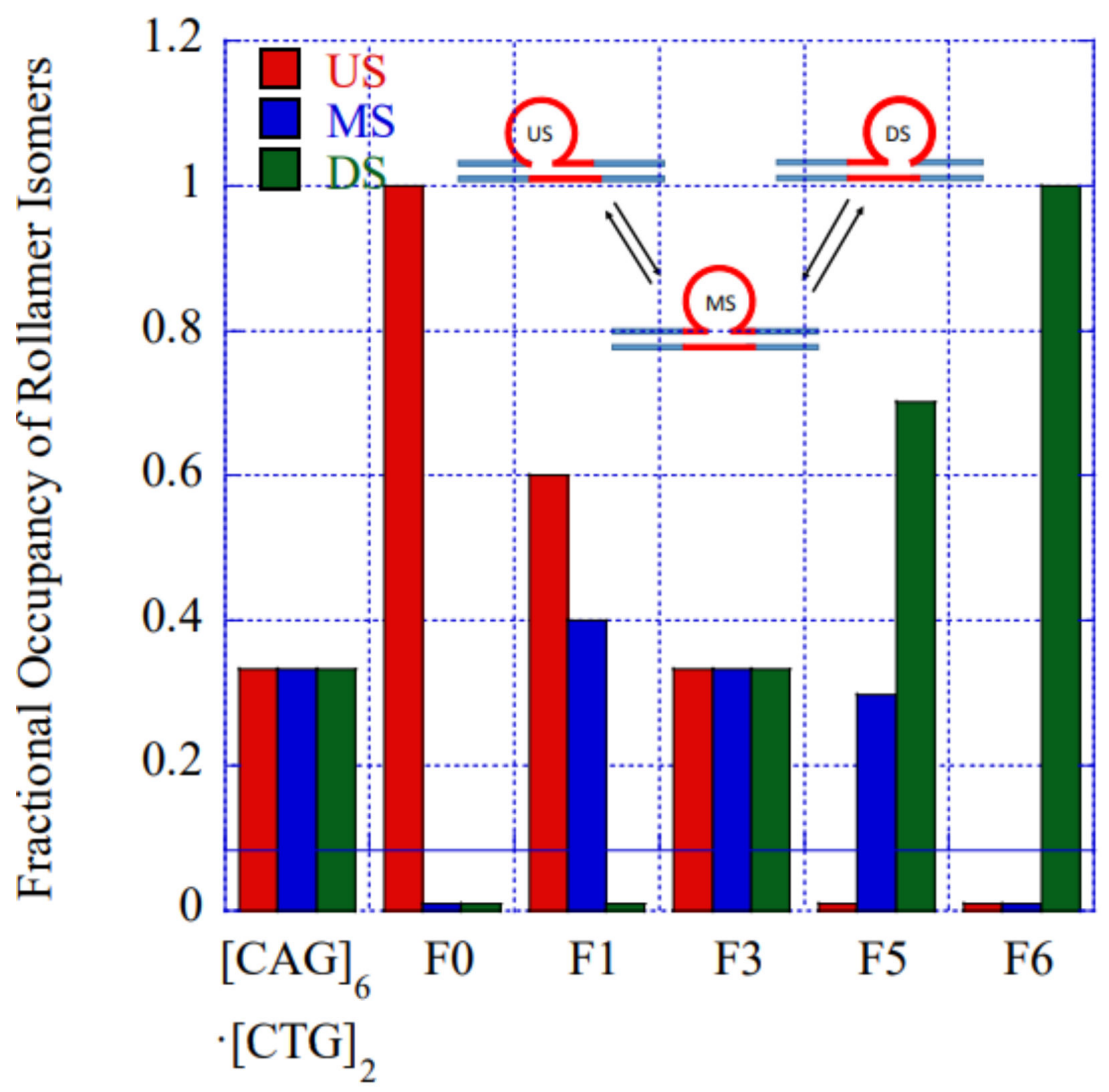
Impact of lesion position on the fractional occupancy of loop isomers at 25 °C derived by fitting to our semi-empirical model. In the absence of lesion induced local changes in the rollamer energy landscape (e.g., in the [CAG]6·[CTG]2 parent or in [CAG]6-F(3)·[CTG]2) all three loop isomers are occupied equally (=0.33). (US = UpStream, MS = MidStream, and DS = DownStream).

To gain further insights into our observations of lesion induced alterations in rollamer distribution, we modeled the measured rollamer excess heat capacity curves. Our model considers the simultaneous presence of three initial states with different fractional occupancies corresponding to the three different rollamer isomers that can be adopted by the [CAG]6-X(n)·[CTG]2 complex. The model includes two melting intermediates corresponding to partially denatured states with either the upstream or the downstream duplex arm and the two proximate CAG·CTG triplets remaining base paired, while the other arm and remaining repeat loop triplets are denatured. This model allows for introduction of a position dependent perturbation to account for the lesions. Many of the initial estimates for the thermodynamic parameters needed to fit our model to the experimental DSC thermograms were extracted from our prior experimental data on the large number of related bulge loop structures that we have studied [32,44,50,60,76]. The values of the fitted parameters, while not necessarily determinative, are plausible within the context of established DNA thermodynamics. A more detailed description of the model will be published elsewhere. Despite its simplicity, the model captures all salient features of the measured excess heat capacity curves for all constructs studied. Importantly, our model allows us to predict fractional occupancies of the individual rollamer loop isomers as a function of lesion position, as depicted in Figure 5. The results shown in this figure complement and reinforce the conclusions derived by our enthalpy analysis described above. To be specific, our model predicts that the presence of the lesion results in a fractional enrichment of those initial loop isomer states that accommodate the lesion with the least energetic perturbation. Less favorable loop isomer states do remain populated, but with significantly reduced fractional occupancies relative to the favorable loop isomer. These modeling results support our hypothesis that the presence of the lesion leads to rearrangement and redistribution of loop isomers within the repeat loop rollamer ensemble, in favor of those rollamer isomers where the lesion impact is least disruptive.

In summary, our experimental and computational results reveal that the formation of a lesion within a triplet repeat domain significantly alters the already complex energy landscape by creating a hierarchy of additional local energy minima within the larger repeat energy landscape (Figure 1). In short, our results demonstrate that lesions can induce second-order alterations in the global, dynamic repeat DNA energy landscape through favorable (lower local energy minima) and unfavorable (higher local energy minima) contributions that influence the ensemble of states that BER enzymes encounter during repair processes; thereby adding an additional level of substrate complexity that likely impacts repair outcomes. Moreover, our most recent results (unpublished data) revealed that such lesion-induced alterations in rollamer landscape and loop distribution extends to systems with more potential rollamer isomers than the three bulge loop isomers described here, consistent with the generality of our conclusions.

### 3.3. Implications of Dynamic Energy Landscapes on DNA Repair

Although oxidative lesion formation can generally be considered as a stochastic process, it has been found that repeat domains are ‘hot spots’ where lesion formation is enhanced relative to other DNA sequences. It has also been suggested that the propensity of triplet repeat domains to adopt, even if only transiently, altered, non-B DNA structures such as bulge loops is one potential reason for enhanced rates of oxidative damage in trinucleotide repeat DNAs.[56] Numerous pathways by which repeat bulge loops might form transiently during replication recombination or repair processes have been proposed [24,77,78,79]. Our in vitro observations reveal that the presence of lesions, even within relatively simple short repeat bulge loop constructs, yield complex dynamic energy landscapes. This result suggests that lesions within larger rollameric repeat bulge ensembles may generate significant challenges for DNA processing machineries in terms of substrate selection. The complexity of the substrate landscape we detected for our relatively simple, well controlled oligonucleotide constructs suggests that within repetitive DNA domains, DNA processing machineries are not only challenged by a multitude of potential substrate conformations, but also by their differential thermodynamic recognition profiles and kinetic availabilities. Our results show that within such rollameric repeat ensembles, a particular lesion, even within the same sequence/ nearest neighbor context and/or sequence position, can adopt multiple conformational states with different occupancy rates; a feature which undoubtedly will impact how effectively DNA processing machineries interact with such substrates.

Consistent with this expectation, Delaney and coworkers have shown reduced OGG1 activity to cleave 8oxodG lesions within CAG repeat structures [56] whereas we have previously shown that APE1 processing is much reduced or completely absent when the abasic site lesion is located within static repeat self-structures [80]. Base excision repair depends on an intricate, tightly controlled handoff of repair intermediates between a group of core proteins, aided by a number of associated helper proteins. [81] Loop redistribution to partition a lesion/repair intermediate into an altered structural domain, and/or the transient occupancy of preferred or inexpedient substrate conformations, would quite likely disrupt the intricate balance of sequential BER repair steps [52,74,75,82,83] necessary for successful repair outcomes, thereby potentially contributing to aberrant expansion events. Intriguingly, the dynamic nature of lesion-induced rollamer distribution may result in either beneficial or detrimental repair outcomes, depending on the context. Our results reported here provide a basis for future testing of the extent to which complex substrate dynamics inhibit or enhance various critical BER steps. For example, we have shown partial recognition and processing/cleavage of abasic sites by the crucial BER enzyme APE1 in rollamer constructs where the abasic site lesion can partition into the 5′ duplex domain (a thermodynamically unfavorable isomer) or the bulge loop (a thermodynamically favorable isomer), whereas the same lesion in a static repeat bulge loop is barely processed [80].The latter observation, not surprisingly, also suggests that APE1 binding/processing can alter loop distribution through its interactions with an unfavorable loop isomer. APE1, however, appears to not be able to process abasic sites where loop rearrangement results in partitioning of the lesion into the loop or 3′ junction domain, both of which are thermodynamically favored lesion positions. In other words, the same lesion within a larger repeat domain can be a suitable substrate for BER repair enzymes or not, depending on the dynamic distribution of repeat bulge loop rollamers and the as yet unknown impact of repair protein binding on loop distribution. We are in the process of evaluating to what extent binding by the repair protein further influences rollamer distributions and thereby repair outcomes.

## 4. Conclusions

We have shown that lesion-induced alterations in the energy landscape of repeat DNAs result in dynamic redistributions of repeat bulge loops so as to minimize the energetic impact of the DNA damage. This dynamic ensemble of potential substrates creates an additional level of complexity with which the DNA processing machinery is confronted. It is not unreasonable to expect that such challenging dynamic substrates contribute to the observed DNA expansion phenomenon upon BER lesion repair. We propose that such conformational fluidity influences repair efficiencies and outcomes and contributes to the processes that lead to DNA expansion.

## Figures and Tables

**Table 1:** Position-dependent, lesion (X)-induced impact on rollamer distributions in [CAG]6-X(n)·[CTG]2*.

Rollamer Constructs	Lesion (x) Position in Sequence	Lesion Positions in Each of the Three Rollamers	Predicted Impacts from Static Loop Studies			Observed Impact on Rollamer Loop Distributions Assessed Calorimetrically and Computationally
			Upstream	Midstream	Downstream	
[CAG]6·[CTG]2 Lesion-free control	N/A	N/A	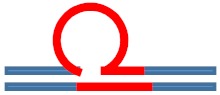	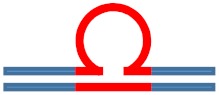	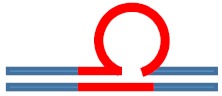	N/A
[CAG]6-X(Stem) ·[CTG]2	Upstream Stem	5 b. upstream 8 b. upstream 11 b. upstream	s. destabilizing - 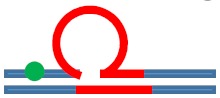	s. destabilizing – 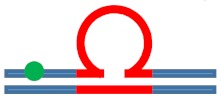	s. destabilizing - 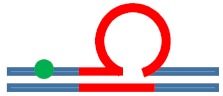	No impact on loop distribution
[CAG]6-F(0) ·[CTG]2	Last base before 1st repeat	5′ junction 3b. upstream 6 b. upstream	m. Destabilizing - 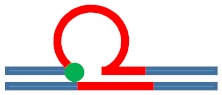	s. Destabilizing - 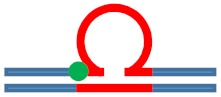	s. Destabilizing - 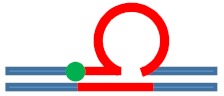	Populates rollamer with lesion at 5′ duplex/loop junction
[CAG]6-X(1) ·[CTG]2	G in 1st repeat	1 repeat loop 5′ junction 3 b. upstream	Neutral 0 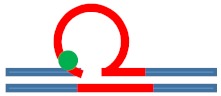	m. Destabilizing - 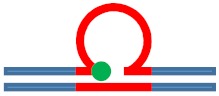	s. Destabilizing -- 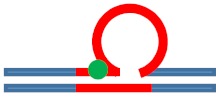	Populates rollamer with lesion within loop
[CAG]6-X(3) ·[CTG]2	G in 3rd repeat	5′ of loop apex Loop apex 3′ of loop apex	Neutral 0 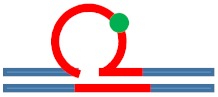	m. Stabilizing + 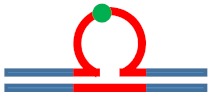	neutral 0 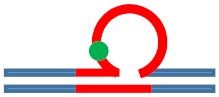	Populates rollamer with lesion within loop
[CAG]6-X(5) ·[CTG]2	G in 5th repeat	3 b. downstream 3′ junction 3 b. into loop	Destabilizing - 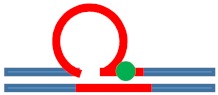	s. Stabilizing ++ 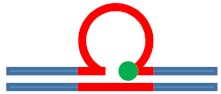	Neutral 0 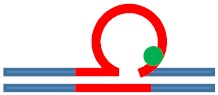	Populates rollamer with lesion at 3/loop/duplex junction
[CAG]6-F(6) ·[CTG]2	G in 6th repeat	6 b. downstream 3 b. downstream 3′ junction	s. Destabilizing - 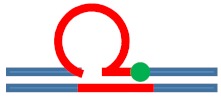	s. Destabilizing - 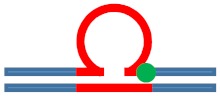	s. Stabilizing ++ 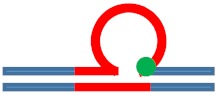	Populates rollamer with lesion at 3/loop/duplex junction

Abbreviations used in table: X—generic placeholder for X = tetrahydrofuran abasic site analogue (F), or X = 8oxodG lesion (O); 3b, 5b number of bases separating lesion position and bulge loop junction; s.—strongly (stabilizing/destabilizing); m.—moderately (stabilizing/destabilizing).

## Supplementary Materials

The following are available online at https://www.mdpi.com/2218-273X/9/11/709/s1, Figure S1: Differential Scanning Calorimetry (DSC) thermograms of static CAG repeat bulge loops containing either an abasic site or a 8oxodG lesion in different sequence positions; Table S1: The impact of the position of the abasic site lesion on static CAG repeat bulge loop transition enthalpy changes; Table S2: The impact of the position of the abasic site lesion on the transition enthalpy changes of the three isomeric, dynamic CAG repeat bulge loop rollamer ensembles.
