# Supplementary material for: Dynamic DNA Energy Landscapes and Substrate Complexity in Triplet Repeat Expansion and DNA Repair
Source: Biomolecules. 2019 Nov 6;9(11):709. doi: 10.3390/biom9110709 (PMC6920812; doi:10.3390/biom9110709)
Supplement: Supplementary file 1 [file biomolecules-09-00709-s001.pdf]

Supporting Information for:

DYNAMIC DNA ENERGY LANDSCAPES AND SUBSTRATE COMPLEXITY IN TRIPLET REPEAT EXPANSION AND DNA REPAIR

Jens Völker[1], G.Eric Plum[2], Vera Gindikin[1], and Kenneth J. Breslauer[1,3]*.

[1] Department of Chemistry and Chemical Biology, Rutgers University, Piscataway, NJ 08854; [2] IBET Inc, Columbus, OH 43220; [3] The Rutgers Cancer Institute of New Jersey, New Brunswick, NJ 08901.

Table of Contents:

1) **Figure S1:** DSC thermograms of static CAG repeat bulge loops containing either an abasic site or a 8oxodG lesion in different sequence positions

2) **Table T1:** The impact of the position of the abasic site lesion on static CAG repeat bulge loop transition enthalpy changes

3) **Table T2:** The impact of the position of the abasic site lesion on the transition enthalpy changes of the 3 isomeric, dynamic CAG repeat bulge loop rollamer ensembles

Figure S1: Excess heat capacity curves measured for the $[CAG]_6$-X(n)·$[CTG]_0$ constructs (where X=F= Tetrahydro Furan abasic site analogue and X=O= 8oxodG) for different lesions positions within the repeat domain X(n), where X replaces the dG in the nth CAG repeat counting from the 5'end. The lesion free parent construct (black) is the same in all panels; blue curves correspond to abasic site (F) lesions; red curves correspond to 8oxodG lesions. A schematic representation of the different lesion positions (green dot) relative to the loop is shown as an insert in each panel. The data presented in Figure S1 complement the rollamer data presented in Figure 4 in the main manuscript and provide the baseline against which the lesion containing rollamer excess heat capacity curves are compared.

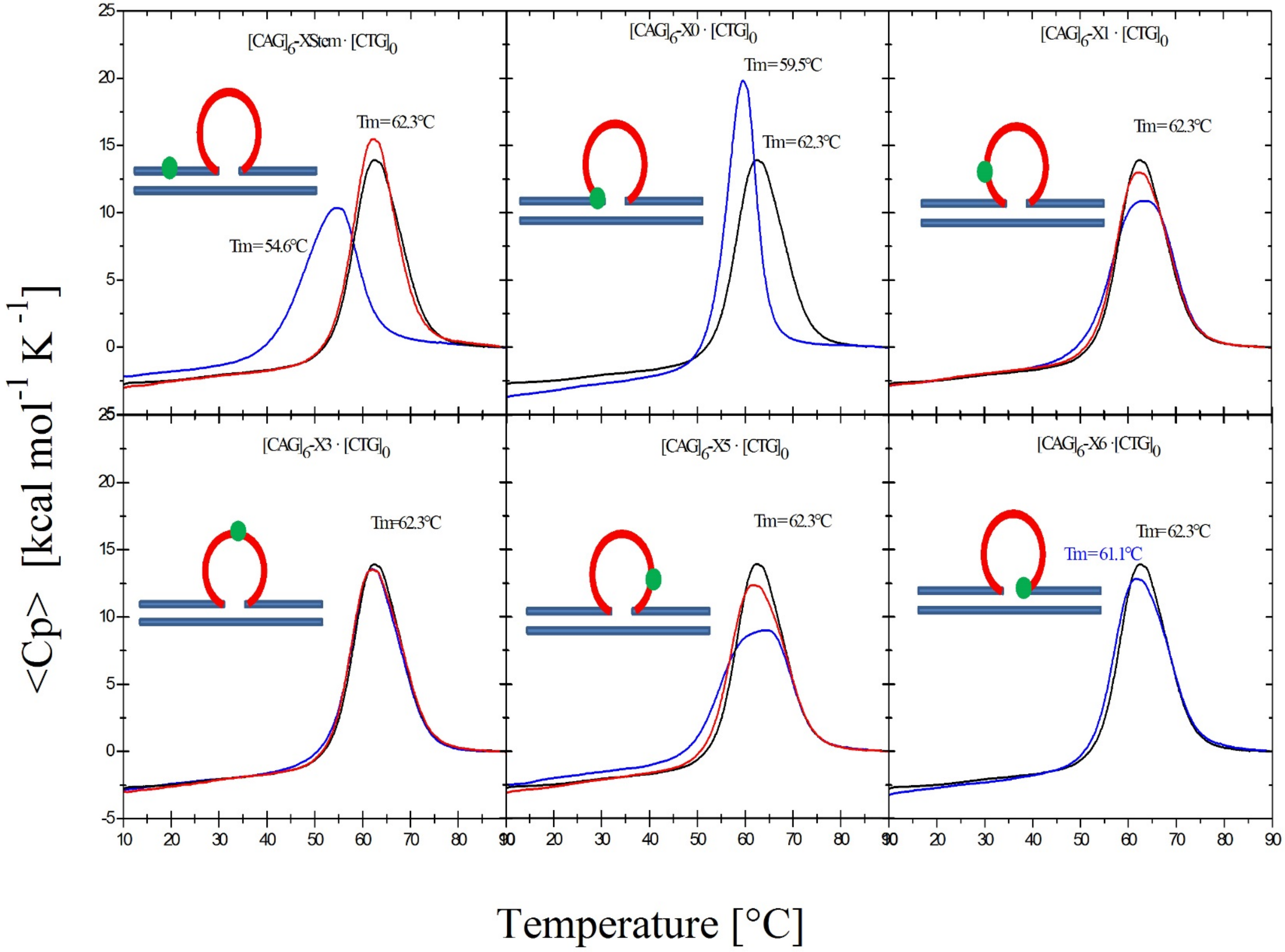
[CAG]6-XStem · [CTG]0
Tm=62.3°C
Tm=54.6°C
[CAG]6-X0 · [CTG]0
Tm=59.5°C
Tm=62.3°C
[CAG]6-X1 · [CTG]0
Tm=62.3°C
[CAG]6-X3 · [CTG]0
Tm=62.3°C
[CAG]6-X5 · [CTG]0
Tm=62.3°C
[CAG]6-X6 ·[CTG]0
Tm=62.3°C
Tm=61.1°C
<Cp> [kcal mol-1 K-1]
Temperature [°C]

Table T1: Thermodynamic parameters: Static CAG Bulge Loop, 6 CAG Repeat Loop, fixed loop position, abasic site varies

Rollamer Construct	Lesion position	Tm [°C]	ΔH_{cal} [kcal mol^{-1}]	$\Delta\Delta H_{cal}$ [kcal mol^{-1}]	IMPACT
$[CAG]_6 \cdot [CTG]_0$	*N/A*	*62.3 ± 0.3*	*173.0 ±8.6*	*0*	*N/A*
$[CAG]_6$-F(Stem) $\cdot[CTG]_0$	*Upstream Stem*	*54.1 ± 0.3*	*146.5 ± 7.3*	*-26.5*	*Destabilizing (--)*
**$[CAG]_6$-F(0)$\cdot[CTG]_0$**	**Last base before 1st repeat**	**59.5 ± 0.3**	**166.0 ± 8.3**	**-7.0**	**Marginally destabilizing (-)**
$[CAG]_6$-F(1)$\cdot[CTG]_0$	*G in 1st repeat*	*62.5 ± 0.3*	*174.1 ± 8.7*	*+1.1*	*Neutral (0)*
$[CAG]_6$-F(3)$\cdot[CTG]_0$	*G in 3rd repeat*	*61.8 ± 0.3*	*173.7 ±8.7*	*+0.7*	*Neutral (0)*
$[CAG]_6$-F(5)$\cdot[CTG]_0$	*G in 5th repeat*	*63.5 ± 0.3*	*148.7 ± 7.4*	*-24.3*	
**$[CAG]_6$-F(6)$\cdot[CTG]_0$**	**G in 6th repeat**	**61.2 ± 0.3**	**183.3 ± 9.2**	**+10.3**	**Stabilizing (+)**

Table T1 lists the melting temperatures and enthalpies we obtained for different lesion positions in the static repeat bulge loop construct derived from the excess heat capacity curves shown in Figure S1. Note we use the difference in enthalpy change relative to the lesion free parent to yield an empirical metric to judge whether the lesion in a given position is overall stabilizing (+), destabilizing (-), or has no impact (0) rather than interpreting the enthalpy in a conventional manner. Such coarse grain metric allows us to score lesion impact in other, more complex repeat bulge loop constructs, such as rollamers with different loop sizes that melt at different temperatures and by more complex pathways, without the need for error prone extrapolations and assumptions. The entrees highlighted in orange/italic in Table T1 have been published by us earlier and are listed to help the reader better understand the rollamer data below. [1] The entrees in black are heretofore unpublished data we collected to complete the database for critical lesion positions in static repeat bulge loop constructs. As discussed previously, the enthalpy for the abasic site containing $[CAG]_6$-F(5)$\cdot[CTG]_0$ construct displays an unexpected low enthalpy coupled with an altered shape of the DSC curve and pre-transition baseline. This experimental result may, in part, reflect interactions between the lesion and the less stable downstream duplex arm, and/or lesion position specific effects that are not yet well characterized. It is listed here for completion and for comparison to the rollamer state, but not considered in our overall analysis metric.

Table T2: Thermodynamic parameters: CAG rollamer, 4 CAG Repeat Loop, 3 possible loop arrangements, abasic site varies

**Rollamer Construct**	**Lesion position**	Abasic Lesion positions in each of the 3 rollamers	**Tm [°C]**	**ΔH_{cal} [kcal mol^{-1}]**	**$\Delta\Delta H_{cal}$ [kcal mol^{-1}]**
**$[CAG]_6 \cdot[CTG]_2$**	**---**	N/A	**75.9 ± 0.3**	**203.8 ± 10.2**	**0**
**$[CAG]_6$-F(Stem) $\cdot[CTG]_2$**	**Upstream Stem**	5 b. upstream<br>8 b. upstream<br>11 b. upstream	**69.3 ± 0.3**	**184.3 ± 9.2**	**-19.5**
**$[CAG]_6$-F(0)$\cdot[CTG]_2$**	**Last base before 1st repeat**	5’ junction<br>3b. upstream<br>6 b. upstream	**69.1 ± 0.3**	**195.0 ± 9.8**	**-8.8**
**$[CAG]_6$-F(1)$\cdot[CTG]_2$**	**G in 1st repeat**	1 repeat loop<br>5’ junction<br>3 b. upstream	**71.5 ± 0.3**	**203.7 ± 10.2**	**-0.1**
**$[CAG]_6$-F(3)$\cdot[CTG]_2$**	**G in 3rd repeat**	5’ of loop apex<br>Loop apex<br>3’ of loop apex	**76.3 ± 0.3**	**197.9 ± 9.9**	**-5.9**
**$[CAG]_6$-F(5)$\cdot[CTG]_2$**	**G in 5th repeat**	3 b. downstream<br>3’ junction<br>3 b. into loop	**76.0 ± 0.3**	**200.8 ± 10.0**	**-3.0**
**$[CAG]_6$-F(6)$\cdot[CTG]_2$**	**G in 6th repeat**	6 b. downstream<br>3 b. downstream<br>3’ junction	**76.1 ± 0.3**	**202.0 ± 10.1**	**-1.8**

Table T2 lists the melting temperatures and enthalpies we obtained for the 3 isomeric rollamer repeat bulge looped constructs containing lesions in different sequence domains. The data are derived from the excess heat capacity curves shown in Figure 4 in the main text. Because the rollamer can adopt 3 different loop positions with different fractional occupancies, the abasic site can partition into different structural domains with corresponding different enthalpic contributions. The observed net enthalpy change consequently reflects the

weighted sum of these three contributions and is, on average, smaller than that seen in the static bulge loop constructs. For example, in $[CAG]_6$-F(0)·$[CTG]_2$ the lesion may partition into the upstream duplex arm for which the static loop data in table T1 suggest a very large negative impact [1]. It also can partition into the 5' loop junction, where the data on static loop listed in table T1 suggests a reduced but still negative enthalpic impact. The observed enthalpy change is moderately negative, roughly of the same magnitude as seen for the 5' junction lesion in the static loop construct in Table T1, suggesting that the fractional occupancy of the isomer with the lesion partitioned in the duplex domain can only be very small. Note that our analysis only takes into account the presence of the lesion in a globally defined structural element. Any subtle effect, such as minor differences in enthalpy change related to the distance/ number of base pairs between the abasic site in the duplex domain and the bulge loop position is outside the scope of our measurement. We also do not yet consider in our analysis potential minor differences in enthalpic impact due to varying lesion position within the loop.

References

1. Volker, J.; Plum, G.E.; Klump, H.H.; Breslauer, K.J. DNA repair and DNA triplet repeat expansion: the impact of abasic lesions on triplet repeat DNA energetics. *Journal of the American Chemical Society* **2009***, 131*, 9354-60, DOI 10.1021/ja902161e.
